# Molecular Therapeutic Advances in Personalized Therapy of Melanoma and Non-Small Cell Lung Cancer

**DOI:** 10.3390/jpm2020035

**Published:** 2012-04-10

**Authors:** Fergal C. Kelleher, Benjamin Solomon, Grant A. McArthur

**Affiliations:** Divisions of Cancer Medicine/Cancer Research, Peter MacCallum Cancer Centre, Locked Bag 1A'Beckett St Victoria 8006, Australia; E-Mails: fergal.kelleher@petermac.org (F.C.K.); ben.solomon@petermac.org (B.S.)

**Keywords:** targeted therapy, non-small cell lung cancer, melanoma, EML4-ALK, BRAF

## Abstract

The incorporation of individualized molecular therapeutics into routine clinical practice for both non-small cell lung cancer (NSCLC) and melanoma are amongst the most significant advances of the last decades in medical oncology. In NSCLC activating somatic mutations in exons encoding the tyrosine kinase domain of the Epidermal Growth Factor Receptor (EGFR) gene have been found to be predictive of a response to treatment with tyrosine kinase inhibitors (TKI), erlotinib or gefitinib. More recently the EML4-ALK fusion gene which occurs in 3–5% of NSCLC has been found to predict sensitivity to crizotinib an inhibitor of the anaplastic lymphoma kinase (ALK) receptor tyrosine kinase. Similarly in melanoma, 50% of cases have BRAF mutations in exon 15 mostly V600E and these cases are sensitive to the BRAF inhibitors vemurafenib or dabrafenib. In a Phase III study of advanced melanoma cases with this mutation vemurafenib improved survival from 64% to 84% at 6 months, when compared with dacarbazine. In both NSCLC and melanoma clinical benefit is not obtained in patients without these genomic changes, and moreover in the case of vemurafenib the therapy may theoretically induce proliferation of cases of melanoma without BRAF mutations. An emerging clinical challenge is that of acquired resistance after initial responses to targeted therapeutics. Resistance to the TKI’s in NSCLC is most frequently due to acquisition of secondary mutations within the tyrosine kinase of the EGFR or alternatively activation of alternative tyrosine kinases such as C-MET. Mechanisms of drug resistance in melanoma to vemurafenib do not involve mutations in BRAF itself but are associated with a variety of molecular changes including RAF1 or COT gene over expression, activating mutations in RAS or increased activation of the receptor tyrosine kinase PDGFRβ. Importantly these data support introducing re-biopsy of tumors at progression to continue to personalize the choice of therapy throughout the patient’s disease course.

## 1. Introduction

The apparently disparate malignant entities of non-small cell lung cancer and melanoma have in recent years emerged as the forerunners in the personalized management of malignant disease. The evolving therapeutic paradigm for these cancers is increasingly guided by an improved understanding of the molecular basis of the diseases and the availability of appropriate targeted therapeutics. In non-small cell lung cancer (NSCLC), tumors with activating mutations of the EGFR have proved to be exquisitely sensitive to treatment with the EGFR tyrosine kinase inhibitors gefitnib and erlotinib. Another subset of tumors molecularly defined by the presence of rearrangements in the anaplastic lymphoma kinase (ALK) gene is similarly susceptible to treatment with crizotinib an oral inhibitor of ALK. The prevailing pessimism in treating metastatic melanoma has recently been dramatically overturned. Previously the only U.S. Food and Drug Administration (FDA) approved systemic treatments in this setting were dacarbazine and high dose interleukin-2, though neither have been demonstrated to confer an overall survival advantage. Vemurafenib, an oral inhibitor of BRAF V600E mutated melanoma which occurs in approximately 50% of cases does however confer a survival advantage. Together NSCLC and melanoma are leading the charge in personalizing care in patients with advanced cancer.

## 2. Molecular Therapeutics and Non-Small Cell Lung Cancer

Each year over 1.6 million patients are diagnosed and 1.3 million die from lung cancer making this the most frequent and most deadly malignancy [1]. Approximately 85% of these cases will be classified as non-small lung cancer (NSCLC), a heterogeneous group of tumors comprised of three major histologic subtypes namely adenocarcinoma, squamous cell carcinoma and large cell carcinoma. Cigarette smoking is the largest single cause of lung cancer, responsible for about 85% of cases in men and 47% of cases in women, worldwide [2]. However, lung cancer in never smokers is a frequent clinical entity which when considered in its own right is the seventh most common cause of cancer related death worldwide [2].

**Figure 1:**
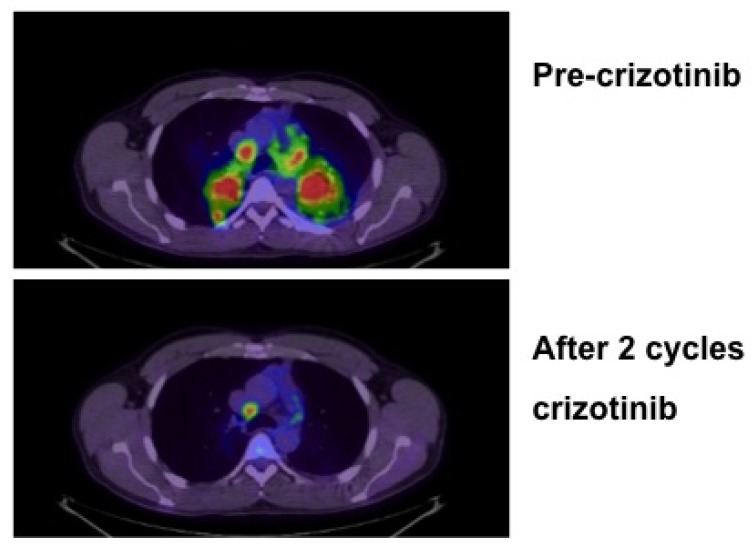
FDG PET/CT scans in a 43 y.o. male never smoker with anaplastic lymphoma kinase (ALK)-positive non-small cell lung cancer (NSCLC) prior to and after two cycles of Crizotinib.

Adenocarcinoma, previously the most common histological subtype of NSCLC only in women and non-smokers, is now more frequent than squamous cell and large cell carcinoma in both males and females [3]. Molecular characterization of lung adenocarcinoma have led to the correlation of activating mutations in the Epidermal Growth Factor Receptor (EGFR) with sensitivity to the EGFR tyrosine kinase inhibitors (TKI) gefitinib and erlotinib and also the identification of rearrangements of the ALK gene in distinct subsets of NSCLCs that are highly sensitive to the ALK inhibitor Crizotinib (Figure 1). These findings linking tumor genotype with clinically available therapeutic agents have brought personalized therapy to the realm of standard therapy for NSCLC.

The first selective orally available EGFR TKIs gefitinib and erlotinib were first used over 10 years ago prior to the discovery of EGFR mutations. Initial clinical trials with gefitinib revealed response rates in the order of 10–20% with minimal toxicity [4,5]. However, a Phase III placebo-controlled study with gefitinib in previously treated NSCLC patients failed to demonstrate a survival benefit [6] and a Phase III study with erlotinib demonstrated only a modest survival benefit [7].

It was however recognized that responses to EGFR TKIs, which were often dramatic and durable, more frequently occurred in certain populations namely females, non-smokers, patients of East Asian and those with adenocarcinoma histology. The basis for the sensitivity of this subset of patients was revealed in 2004 when somatic mutations in the EGFR tyrosine kinase domain were described [8,9,10]. The most common activating mutations are deletions in exon 19 and the L858R point mutation in exon 21 that account for approximately 85% of all activating EGFR mutations. Less frequent activating mutations including G719S in exon 18 and L861Q in exon 21 have also been described. Prospective studies of EGFR TKIs in patients with EGFR mutations have consistently identified high response rates and prolonged progression free survival. The Spanish Lung Cancer Group screened 2105 patients, to identify 350 patients (15%) with exon 19 deletions and L858R of whom 250 patients went on to receive erlotinib in the first or second-line setting. In this population, the response rate was 70% with median time to progression of 14 months and median overall survival of 27 months [11]. Six randomized studies have now been reported that demonstrate the superiority of either gefitinib or erlotinib over chemotherapy in NSCLC patients with EGFR mutations [12,13,14,15,16,17]. In one of these studies, the IPASS study [12]; patients with clinical features associated with EGFR mutations were randomized to receive either gefitinib or chemotherapy with carboplatin and paclitaxel. Those with EGFR mutations had a significantly increased response rate (71.2% *vs.* 47.3%) and progression free survival (HR = 0.48, 0.36–0.64, p < 0.001) with gefitinib compared with chemotherapy. Notably, in this population with clinical features associated with response to EGFR TKIs, patients without EGFR mutations did significantly worse with geftinib than they did with chemotherapy (median PFS 1.6 months compared with 5.5 months, HR 2.85, p < 0.0001) confirming the importance of selecting therapy based on genotype rather than clinical features. Together these six Phase III studies firmly establish a role for EGFR mutation testing in selecting optimal therapy for newly diagnosed patients with advanced NSCLC [18].

A second molecular driver that has been identified and effectively targeted in NSCLC is the *ALK* gene. Originally identified in an uncommon T-Cell lymphoma called anaplastic large cell lymphoma, rearrangements in the *ALK* gene were first identified in NSCLC in 2007 [19,20]. These typically arise through a small inversion in chromosome 2 that results in a fusion gene (*EML4-ALK*) that encodes a chimeric protein with an N-terminal derived from Echinoderm microtubule associated protein like 4 and the C-terminal containing the tyrosine kinase domain of ALK that has aberrant cytoplasmic expression and constitutive activity. Different *EML4-ALK* variants containing variable portions of the *EML4* gene consistently fused to exon 20 of the *ALK* gene have been identified as have other rare fusion partners including *TFG* and *KIF5B*. Preclinical data indicate that *ALK* rearrangements were transforming both *in vitro* and *in vivo* and conferred sensitivity to ALK inhibitors indicating their potential as a therapeutic target. Subsequent studies have shown that these rearrangements are uncommon, occurring in approximately 3–4% of NSCLC but seem to occur most frequently in younger patients (median age 51), never or light smokers and those with adenocarcinoma histology [21]. These rearrangements are almost always mutually exclusive with *EGFR* mutations or *KRAS* mutations. In a Phase I first in class trial of the orally available ALK TKI crizotinib (Xalkori, Pfizer) an Objective Response Rate of 61% with an estimated median Progression free survival of 10 months was reported in a cohort of 116 patients with ALK-positive NSCLC identified by Fluorescence in situ hybridization (FISH) using break-apart probes [22,23]. The drug was in general well tolerated with frequent but generally mild nausea, vomiting, diarrhea, and transient visual disturbances accounting for the majority of toxicities. Based on its efficacy as well as safety, crizotinib was granted accelerated approval by the FDA in August of 2010 for the treatment of patients with advanced ALK-positive NSCLC. Two Phase III studies, in the first line and the second line comparing crizotinib to standard chemotherapy are ongoing ((NCT00932893 and NCT01154140). While there are several methods that have been used to detect *ALK* rearrangements in tumor tissue including immunohistochemistry, fluorescent *in-situ* hybridization (FISH) and reverse transcriptase polymerase chain reaction, FISH testing was used as a companion diagnostic to identify patients for the crizotinib trials [22] and has emerged as the gold standard method to identify *ALK* gene rearrangements and is the only FDA approved test for this purpose. 

In addition to *EGFR* mutations and *ALK* rearrangements, genomic studies have also identified frequent copy number changes and somatic mutations affecting components of key signaling pathways in adenocarcinoma of the lung including *KRAS, Her2, BRAF, C-MET, MEK1 and PIK3CA* [24,25] (Table 1). What is notable is that many of these oncogenic drivers are targetable by compounds that are either approved or at varying stages of clinical development. BRAF mutations, for instance, are found in approximately 3% of adenocarcinoma of the lung [26]. They tend to occur in smokers with adenocarcinoma and in contrast to melanoma where over 90% of mutations are V600E, in lung cancer only about 50% are V600E (usually G469A and D594G). As has proven to be the case in V600E *BRAF* mutant melanoma there is a compelling rationale for targeting this population of patients with BRAF inhibitors. Mutations within exon 20 that encodes part of the kinase domain of *Her2* have been reported with a frequency of approximately 2–4% and tend to occur in never smokers with adenocarcinoma [27,28,29]. These mutations may be sensitive to treatment with traztuzumab or with panHer inhibitors such as dacomitinib (PF299804, Pfizer) or afatinib (BIBW 2992, Bohringer-Ingelheim). *KRAS* mutations are reported in 15–25% of patients with lung adenocarcinoma and are typically mutually exclusive with *EGFR* mutations or *ALK* gene rearrangements. They are more common in smokers, less common in East Asian populations, and are associated with resistance to EGFR TKIs [30,31]. While KRAS has long been considered “undruggable” and no drugs that specifically target KRAS have been developed to date, strategies to target KRAS by inhibiting both PI3 Kinase and MAP Kinase signaling pathways are under clinical evaluation.

**Table 1:** Major molecular subtypes of non-small cell lung cancer. Frequency of molecular subtypes are derived from references in the text together with the Catalogue of Somatic Mutations in Cancer (COSMIC) (http://www.sanger.ac.uk/genetics/CGP/cosmic/, accessed on 20 November 2011) and MyCancerGenome.org (http://www.mycancergenome.org/ accessed on 20 November 2011).

Histologic Subtype	Molecular Sub Category (Frequency)
Adenocarcinoma (~50% of NSCLC)	
	EGFR tyrosine kinase domain mutations (10–35%)
	KRAS mutations (15–25%)
	ALK Gene Rearrangements (3–5%)
	Her2 mutations
	BRAF mutations (3%)
	MET amplification (1%)
Squamous Cell Carcinoma (~30% of NSCLC)	
	FGFR1 Amplification (20%)
	DDR2 mutation (4%)
	PIK3CA mutations (1–3%)
	EGFRvIII mutations (<5%)

In contrast to the frequent occurrence of potential therapeutic targets in adenocarcinoma, particularly occurring in nonsmokers, there was a paucity of identified molecular targets in tumors from smokers with squamous cell histology. Recently, however, several potentially targetable genes have been identified in squamous cell carcinoma of the lung. Focal amplification of the Fibroblast Growth Factor Receptor 1 (*FGFR1*) gene has recently been identified in up to 20% of squamous cell carcinomas [32,33]. Tumors with *FGFR1* amplification may be dependent of fibroblast growth factor receptor (FGFR) signaling for survival and are sensitive to FGFR inhibitors. Several potent FGFR inhibitors are in clinical development to target this population (e.g., BGJ398 (Novartis) and AZD4547 (AstraZeneca)). Mutations have also been identified in the discoidin domain receptor 2 (*DDR2)* tyrosine kinase gene in 3.8% (11/290) of SCC samples [34] which may potentially be sensitive to treatment with dasatinib. Other mutations such as those involving the extracellular domain of the *EGFR* (*EGFRvIII*) [35] or PIK3CA [36] have also been described in squamous cell carcinoma and are potentially amenable to therapeutic intervention.

## 3. Melanoma

Melanoma is the subtype of skin cancer associated with the highest mortality, being the fourth most common cancer in Australia and had an estimated incidence of 70,000 cases in the US in 2011. Until recently there were only two drugs, interleukin-2 and dacarbazine, that had been approved by the US FDA for the treatment of metastatic melanoma. Responses to these therapeutics are achieved in 10–20% of patients however there are no randomized trials demonstrating an overall survival advantage. Recent efforts to search for molecular drivers of melanoma have identified frequent aberrations in a number of well characterized oncogenes and tumor suppressor genes including: BRAF (50–60% mutated), NRAS (15–20% mutated), AKT3 over expression, CDKN2A (30–70% deleted, mutated, or silenced), PTEN (5–20% deleted or mutated), APAF1 (40% silenced), TP53 (10% lost or mutated) with amplification of genes such as CCND1 and MITF occurring at varying frequencies [37]. The most frequently mutated genes in melanoma with their respective frequencies of mutation are listed in Table 2. One study of 102 melanomas found mutations or increased copy number of KIT in 28% of primary melanomas arising from chronic sun damaged skin, 36% of acral and 38% of mucosal melanomas [38]. The correlative implicated genes depending on anatomic site that a melanoma arises from and the type of sun exposure are documented in Table 3. KIT has emerged as an important oncogene in melanoma that has been targeted with KIT inhibitors such as imatinib, nilotinib or dasatinib. Therefore the tumor biology is well described and additional molecularly directed treatments are likely to emerge in the future.

**Table 2:** Genes most frequently mutated in melanoma. Data from COSMIC database, (accessed on 20 November 2011).

Gene	Frequency (%)	Gene	Frequency (%)
BRAF	45	GNAQ	8
CDKN2A	29	CTNNB1	6
NRAS	19	NF2	5
TP53	17	PDGFRA	4
PTEN	17	PIK3CA	2
STK11	10	HRAS	2
FGFR2	9	KRAS	2
KIT	8	GNA11	2

**Table 3:** Major molecular subtypes of melanoma. Source data references [38,47,48,50].

Melanoma	Implicated genes (approximate frequencies)
Arising from skin without chronic sun damage	Mutant BRAF (59%), mutant RAS (22%) KIT mutations or increased copy number (0%)
Arising from skin with chronic sun damage	Mutant BRAF (11%), mutant RAS (15%), mutant or increased copy number KIT (28%)
Arising from mucosal surfaces	Mutant BRAF (11%), mutant RAS (5%) mutant or increased copy number KIT (38%)
Arising from acral surfaces	Mutant BRAF (23%), mutant RAS (10%) mutant or increased copy number KIT (36%)
Uveal melanomas	Mutations in GNAQ or GNA11 (83%)

Activating mutations in the gene encoding the serine-threonine protein kinase BRAF have been observed in 40–60% of melanomas, 40–70% of papillary or anaplastic thyroid cancers and 7–8% of all cancers. The high frequency of substitutions of glutamic acid for valine (V600E) was first recognized by work conducted at the Sanger Institute, in Cambridge, the United Kingdom [39]. 

A Phase I trial was conducted of the BRAF inhibitor vemurafenib in patients with metastatic cancer [40]. There was a dose escalation phase (55 patients, 49 of whom had melanoma) and an extension phase (32 patients with V600E mutant melanoma). The recommended Phase II dose of vemurafenib that emerged was 960mg BD orally and the most frequent toxicities were rash, fatigue and arthralgia. In the dose escalation cohort of the 16 patients with melanoma with the V600E mutation that received 240mg of vemurafenib twice daily or more, 10 had a partial response with 1 complete response. In the extension phase cohort 24 patients had a partial response and 2 had a complete response. 

Squamous cell carcinomas and kerathoacanthomas are a side effect of treatment with vemurafenib that emerges in a subset of patients. This is a phenotypic manifestation of paradoxical activation of the MAPKinase pathway depending on the cellular context, with cells without activating BRAF mutations being activated [41,42,43]. In the Phase I trial squamous cell carcinoma of the keratoacanthoma type was seen in 31% (10 of 32) of patients. These are well differentiated tumors with no cases of clinically significant local invasion or metastases documented to date; however it must be cautioned that the natural history of these cutaneous lesions which arise in this context is still being evaluated and requires further longitudinal and histologic follow up. These secondary tumors are usually excised without a need for treatment interruption.

BRIM-2 was a single arm Phase II open label trial in which 132 patients received vemurafenib until disease progression. Median progression free survival was 6.2 months with a response rate of 52% and a disease stabilization rate of 30%. A succeeding Phase III study the BRIM-3 study was reported upon in 2011. In that study of 675 patients with previously untreated metastatic melanoma, vemurafenib (960mg twice daily orally) was compared with dacarbazine (1000 mg/m2 intravenously every 3 weeks). At 6 months overall survival was 84% and 64% respectively. An interim analysis was undertaken regarding overall survival and a final analysis was performed for progression-free survival. In these assessments vemurafenib was associated with a relative reduction of 63% in the risk of death and of 74% in the risk of either death or disease progression compared to the dacarbazine control arm (P < 0.001, in both cases). The response rates were 48% for vemurafenib and 5% for dacarbazine. In the vemurafenib cohort cutaneous squamous cell carcinomas, kerathoacanthoma or both occurred in 18% of patients. Pathologic analysis of tumor biopsy specimens from these patients may be informative as to whether paradoxical up regulation of the MEK pathway in BRAF wild type cells is the underlying molecular mechanism for these lesions arising. HRAS mutations have been found in kerathoacanthomas and squamous cell carcinomas arising as an iatrogenic manifestation of treatment with vemurafenib [44,45]. This shows how vemurafenib can be beneficial for tumors of one molecular phenotype (V600E mutant) but potentially adverse for another (HRAS/NRAS mutant). 

Molecular therapeutics in melanoma are not just restricted to treatments directed at the MAPK pathway. In a recent Phase II study of 43 patients with metastatic melanoma with KIT aberrations (mutation or amplification) treated with imatinib an overall response rate of 23.3% was observed [46]. Furthermore other molecular therapeutic opportunities exist. In one study of melanomas arising at chronic sun damaged sites CDK4 and CCND1 were implicated as independent oncogenes in melanomas without mutations in BRAF or N-RAS [47].

## 4. Uveal Melanoma

BRAF and NRAS mutations occur frequently in benign and malignant neoplasm’s that arise from melanocytes in epithelial structures. Notably, uveal melanomas arise from melanocytic nests within the uveal tract comprising the choroid, ciliary body and iris. In contrast to cutaneous melanomas uveal melanomas rarely if at all have activating mutations in BRAF. Investigations lead by Boris Bastian of the University of California San Francisco, assessed the frequency of GNAQ mutations in a variety of melanocytic neoplasms following the discovery of frequent GNAQ mutations in ‘malignant blue nevi’ which are intra-dermal proliferations of melanocytes. Mutations in GNAQ were found in 46% of uveal melanomas and 27% of uveal melanoma cell lines [48].

Prompted by similar phenotypes in mice harboring germline mutations in GNAQ and GNA11 [49], GNA11 was also evaluated in a series of uveal melanomas, blue nevi and other nevi. GNA11 was mutated in 7% of blue nevi and 32% of primary uveal melanomas and 57% of metastasis arising from uveal melanomas [50]. Overall, it is important to appreciate those melanomas that arise from melanocytes of differing tissues or origins display unique mutational profiles. This raises the concept that personalized therapy can be driven by the developmental biology of the cell lineage that becomes transformed in cancer. 

## 5. Molecular Drug Resistance and the Emergence of Drug Resistant Tumor Clones

Cancer is a dynamic evolving biological process. Genetic differences are frequently found between primary tumors and their arising secondary metastases and even within primary tumors molecular heterogeneity is often found. The advent of molecularly targeted therapeutics demands vigilance by the treating physician to the phenomenon of clonal evolution and secondary drug resistance and for treatment decisions to be informed not just by an initial histologic diagnosis and genomic profile but also by molecular evaluation when progression occurs by performing re-biopsy of tumors at disease progression. An illustrative historic example is that in chronic myelogenous leukemia which is initially sensitive to treatment with imatinib mesylate (targets the BCR-ABL) treatment resistance may emerge due to the acquisition of secondary mutations in BCR-ABL that are resistant to imatinib but that are sensitive to second generation BCR-ABL inhibitors such as dasatinib or nilotinib. Nilotinib and dasatinib are effective in nearly all such cases with the exception of cases that acquire the T315I mutation.

## 6. Acquired Resistance in NSCLC

Despite initial sensitivity and responses that in many cases can be maintained over periods of many months or even years, tumors with EGFR mutations or ALK gene rearrangements treated with specific inhibitors will ultimately progress—a process termed acquired resistance. In the case of acquired resistance to gefitinib and erlotinib in EGFR mutant tumors two major mechanisms have been identified. Firstly, a secondary mutation, the T790M mutation within exon 20 of EGFR, is responsible for about 50% of instances of acquired resistance [51,52]. This mutation increases the affinity of the EGFR for ATP approximately 10-fold and allows ATP to competitively displace gefitinib and erlotinib from EGFR [53]. Other less common point mutations, such as D761Y, have also been reported which confer acquired resistance to EGFR TKIs. Although irreversible inhibitors of the EGFR such as dacomitinib or afatinib inhibit T790M in vitro their clinical activity in the setting of tumors with T790M mutations remains to be demonstrated and novel mutation specific T790M inhibitors are in clinical development. A second mechanism for resistance which is seen in 5–20% of patients is amplification of CMET which causes resistance to EGFR TKIs by activating PI3 Kinase signaling through ERBB3 [54,55,56,57]. Other mechanisms of resistance including transformation of adenocarcinoma to small cell carcinoma histology indicates the complexity of the genetic or epigenetic changes responsible for the phenomenon of acquired resistance [55]. 

The mechanisms of acquired resistance to crizotinib are only just coming to light but, analogous to acquired resistance to EGFR inhibitors, secondary mutations in ALK that render the ALK kinase resistant to inhibition by crizotinib have been identified [59,60,61]. These mutations including L1196M in the ALK kinase domain may be susceptible to inhibition with novel ALK inhibitors e.g., LDK378, AP26113, or AF802. However, other mechanisms of resistance including activation of alternative kinases e.g., EGFR have also been implicated in resistance to crizotinib [57].

## 7. Acquired Resistance in Melanoma

Despite the impressive response rates observed with vemurafenib in the treatment of metastatic melanoma there are a proportion of patients that do not respond and have a primary refractory state to treatment with the BRAF inhibitor. There is also a phenomenon of secondary acquired drug resistance. For example in the Phase I study an estimated median progression free survival of more than 7 months was observed but ultimately drug resistance emerged in a significant proportion of patients. In non-small cell lung cancers sensitive to EGFR tyrosine kinase inhibition is lost usually due to ‘gatekeeper’ mutation however surprisingly acquisition of BRAF gatekeeper ‘mutations’ do not account for secondary drug resistance in melanoma that acquire resistance to BRAF inhibitors. Two separate high profile studies have been published that evaluated the molecular mechanisms underlying secondary resistance of metastatic melanoma to treatment with vemurafenib. In one study it was shown that acquired resistance to Vemurafenib develops by mutually exclusive PDGFRB up regulation or by NRAS mutations [62]. The second study identified that the gene encoding COT/TpI2 called MAP3K8 is a MAPK pathway activator and maybe responsible for the evolution of resistance to vemurafenib in some cases of V600E mutated melanoma [63] Furthermore it subverts RAF signaling by activating ERK signaling by mechanisms downstream of BRAF that are not RAF dependent. The therapeutic implications of these combined studies are important. For instance there is a putative role for combinatorial therapeutics that target RAF and MEK, the latter acting downstream of BRAF. Additionally targeting of COT and PDGFRβ may subvert BRAF inhibitor *de novo* or secondary drug resistance. Based on these limitations the development of new strategies to inhibit signaling cascades that escape BRAF inhibition, are ongoing. It is interesting to speculate why gatekeeper mutations are not usually found as a cause of molecular drug resistance in melanoma. A speculation is that gatekeeper mutations would most likely reduce the kinase activity of mutant BRAF negating its oncogenic potential. 

## 8. Conclusions

There have been exciting developments in the molecular treatment of two disorders, melanoma and NSCLC which have for decades been characterised by poor outcomes with chemotherapeutic treatment when the disease was at an advanced stage. The rapid transition from scientific findings to clinical application is exemplified by both the finding of BRAF mutations in melanoma in 2002 and FDA approval of vemurafenib for this indication in 2011 and that of the initial finding of EML4-ALK in 2007 and FDA approval of crizotinib in 2011. While, ongoing sequencing efforts continue to uncover new genetic drivers of cancer and continue to uncover new opportunities for the personalized therapy of melanoma and lung cancer with the discovery of new targets and the evaluation of rational combination therapies there remain many challenges in deploying these advances to standard practice. The emergent knowledge of molecular tumor profiles e.g., EGFR and KRAS mutations and ALK rearrangements for instance raises logistic and algorithmic considerations to approaching the molecular management of the adenocarcinoma subtype of NSCLC. Ideally all three abnormalities should be tested simultaneously to provide rapid molecular diagnosis for patient and physician. Economic and technical limitations may demand a sequential or step-wise approach [64]. Ultimately the development of an affordable novel technology to allow extensive profiling of all the possible genomic drivers of a patients cancer is required. Newer sequencing technologies may eventually enable such an approach. The acquisition of acquired resistance after initial effective therapy remains a daunting clinical problem. Unanswered questions include: What is the most effective way to subvert the emergence of molecular drug resistance? Do we require the upfront treatment of disease with combined molecularly targeted therapeutics? Also are there molecular predictors of the likelihood of the emergence of treatment resistance? Huge progress has been made but there remains a long way to go to deliver personalized medicine for all patients with lung cancer and melanoma.
